# Low NCOR2 levels in multiple myeloma patients drive multidrug resistance via MYC upregulation

**DOI:** 10.1038/s41408-021-00589-y

**Published:** 2021-12-04

**Authors:** Tomoaki Mori, Rakesh Verma, Rie Nakamoto-Matsubara, Ka Tat Siu, Cristina Panaroni, Keertik S. Fulzele, Kenta Mukaihara, Chukwuamaka Onyewadume, Allison Maebius, Hiroki Kato, Lai Ping Wong, Ruslan I. Sadreyev, David T. Scadden, Noopur S. Raje

**Affiliations:** 1 Center for Multiple Myeloma, Division of Hematology and Oncology, MGH Cancer Center, Massachusetts General Hospital, Harvard Medical School, Boston, MA 02114 USA; 2 Center for Regenerative Medicine, Massachusetts General Hospital, Boston, MA 02114 USA; 3 Department of Molecular Biology, Simches Research Center, Massachusetts General Hospital, Boston, MA 02114 USA

**Keywords:** Oncogenes, Chemotherapy, Cancer genomics

## Abstract

MYC upregulation is associated with multidrug refractory disease in patients with multiple myeloma (MM). We, isolated patient-derived MM cells with high MYC expression and discovered that NCOR2 was down-regulated in these cells. NCOR2 is a transcriptional coregulatory protein and its role in MM remains unknown. To define the role of NCOR2 in MM, we created NCOR2 knockout human myeloma cell lines and demonstrated that NCOR2 knockout led to high MYC expression. Furthermore, NCOR2 knockout conferred resistance to pomalidomide, BET and HDAC inhibitors, independent of Cereblon (CRBN), indicating high MYC expression as a cause of multidrug resistance. Moreover, NCOR2 interacted with the nucleosome remodeling and deacetylase (NuRD) complex and repressed the expression of CD180 by directly binding to its promoter and inducing MYC expression. Next, we generated lenalidomide-resistant and pomalidomide-resistant human myeloma cell lines. Whole-exome sequencing revealed that these cell lines acquired the same exonic mutations of NCOR2. These cell lines showed NCOR2 downregulation and MYC upregulation independent of CRBN and demonstrated resistance to BET and HDAC inhibitors. Our findings reveal a novel CRBN independent molecular mechanism associated with drug resistance. Low NCOR2 expression can serve as a potential biomarker for drug resistance and needs further validation in larger prospective studies.

## Introduction

The immunomodulatory drug thalidomide, and its analogs, lenalidomide, and pomalidomide (IMiDs) have significantly changed the treatment paradigm of multiple myeloma (MM) [1], but most myeloma patients face multiple relapses due to drug resistance.

Recently, Cereblon (CRBN) has been identified as the primary target of IMiDs [2]. IMiDs bind to CRBN, a substrate adaptor of the CRL4^CRBN^ E3 protein ligase complex that mediates degradation of the target proteins, IKAROS and AIOLOS [3]. Downregulation of IKAROS and AIOLOS induces downregulation of MYC, a protein essential for myeloma proliferation and survival [4, 5]. Upregulation of MYC and loss of CRBN are known to be associated with IMiD resistance and poor prognosis [6], although acquired mutations of CRBN or other genes downstream of CRBN E3 ligase pathway have only been reported in 22% of IMiD refractory patients [7]. These data therefore point to CRBN independent IMiD-resistance mechanisms which may serve as additional mechanisms of drug resistance.

NCOR2 and its homolog NCOR1 are nuclear receptor co-repressors (NCORs), which activate histone deacetylase (HDACs) and alter epigenomic modification [8]. These diverse transcription factors play an oncogenic role [9] including in acute myeloid leukemia and several solid tumors. However, the biological significance of NCOR2 in MM remains to be determined. Here, we report a novel pathway involving NCOR2 mediated drug resistance in myeloma independent of CRBN.

## Materials and methods

### Cell lines and reagents

MM.1 s human MM cells were gifts from Dr. Steven Rosen (Northwestern University, Chicago, IL, USA) and cultured in complete media (RPMI 1640 media supplemented with 10% fetal bovine serum [FBS], 2 mM L-glutamine, 100 unit/ml penicillin, 100 µg/ml streptomycin). CRBN knock out 293 T cell line was kindly provided by the Munshi lab at Dana-Farber Cancer Institute. Lenalidomide, Pomalidomide, ACY1215 and MS275 were obtained from Selleck Chemicals (Houston, TX, USA), and CPI0203 was obtained from Sigma-Aldrich (St Louis, MO, USA). Each of these chemicals were dissolved first in DMSO at a concentration of 10 mM, and then in culture medium before use. BM aspirates from MM patients were obtained after approval from the Massachusetts General Hospital Institutional Review Board. Informed consent was obtained in all cases as per the Declaration of Helsinki. After mononuclear cell separation, MM cells were purified by positive CD138 (Syndecan-1) MicroBead selection, as described previously [10]. Cell lines were tested for *Mycoplasma* negatively before use.

### Establishment of lenalidomide-resistant and pomalidomide-resistant MM.1 s

Lenalidomide resistant MM.1 s (Len-R) and Pomalidomide-resistant MM.1 s (Pom-R) were created by incubating MM.1 S with Lenalidomide or Pomalidomide, gradually dosing up to 100 μM.

### Knockout of NCOR2 using CRISPR-Cas9 Technology

Lentiviral constructs expressing CRISPR-associated protein 9 (Cas9) and guide RNAs (gRNAs) originally generated from Feng Zhang’s lab were obtained from Addgene (Cambridge, MA). We first established MM.1 s stably expressing Cas9 by infection of MM.1 s with lentivirus expressing Cas9. A total of three gRNAs targeting NCOR2 were selected from CRISPR pooled libraries generated from Dr. Brunello Doench, including NCOR2 #1 AGGGATCCCTCGGTCCTACG; NCOR2 #2 GACAGCGCCATCACATACCG; NCOR2 #3 GCTGCTGCAAGATCTCATCG. They were synthesized and cloned into the Lentiguide-puro plasmid (#52963) purchased from Addgene. Lentivirus harboring nontargeting vector (Vec) and all gRNA expression constructs were generated and used to infect MM.1 s at day 3 after infection, puromycin was added to the media (5 μg/ml) in order to select infected cells. The expression of NCOR2 in sorted cells was further evaluated by immunoblotting assay and qPCR.

### Cell viability assay

The effect of Lenalidomide, Pomalidomide, CPI0203, ACY1215 and MS275 on the viability of MM cell lines was assessed using the CellTiter-Glo Luminescent Cell Viability Assay (Promega, Madison, WI, USA) according to the manufacturer’s instructions.

### Real-time PCR analysis

Total RNA from MM cell lines was purified with the RNeasy Mini Kit (QIAGEN) according to the manufacturer’s instructions. Reverse transcription was carried out using Transcriptor Reverse Transcriptase (Life Technologies) with oligo-dT primer. cDNAs were then amplified using GoTaq Green Master Mix (Promega) with a pair of gene-specific primers. Primer sequences for the analyzed genes were as follows:

*NCOR2* forward: 5′-TGCAGATCATCTACGACGAGA-3′;

*NCOR2* reverse: 5′-TCCGCATCGCCTGGTTTATTT-3′;

*MYC* forward: 5′-CCCTCCACTCGGAAGGACTA-3′;

*MYC* reverse: 5′-GCTGGTGCATTTTCGGTTGT-3′;

*CRBN* forward: 5′-CAGTCTGCCGACATCACATAC-3′;

*CRBN* reverse: 5′-GCACCATACTGACTTCTTGAGGG-3′;

*AR* forward: 5′-CCAGGGACCATGTTTTGCC-3′;

*AR* reverse: 5′-CGAAGACGACAAGATGGACAA-3′;

*BARD1* forward: 5′-CTGCTCGCGTTGTACTAACAT-3′;

*BARD1* reverse: 5′-TCCAATGCAGTCACTTACACAAT-3′;

*MAX* forward: 5′-CCTGGGCCGTAGGAAATGAG-3′;

*MAX* reverse: 5′-GTCAGCCGCAGATTGAAACC-3′;

*CRMP1* forward: 5′-AGTGACCGACTCCTCATCAAA-3′;

*CRMP1* reverse: 5′-CCAGGAACGATTAAGTTCTCTCC-3′;

*KRTAP4-12* forward: 5′-CTCTGTGTGCTCTGACCAGG-3′;

*KRTAP4-12* reverse: 5′-GTGCCTCCTCTTGCTGCTAA-3′;

*PABPC1* forward: 5′-CTTGCCTCGCTTTACGTGG-3′;

*PABPC1* reverse: 5′-GAAGTTGATGTAGGCGTAGCC-3′;

*UBC* forward: 5′-CTGGAAGATGGTCGTACCCTG-3′;

*UBC* reverse: 5′-GGTCTTGCCAGTGAGTGTCT-3′;

*GAPDH* forward: 5′-ACTGTGGATGGCCCCTCCGG-3′;

*GAPDH* reverse: 5′-GCAGCGCCAGTAGAGGCAG-3′;

### Co-immunoprecipitation (coIP)

The Pierce™ classic magnetic IP/Co-IP kit (Thermo Fisher Scientific, #88804) was used for co-immunoprecipitation of NCOR2 in MM.1 s cells. The lysed cells were transferred to tubes and subjected to 20 min centrifugation at 13,000 × *g*. The supernatant was transferred into a fresh tube. Five hundred ug protein from each sample was diluted to 500 ul and mixed with 5 ul NCOR2 antibody (Abcam, ab24551) or 5 ul normal mouse IgG (Sigma-Aldrich) as a negative control and incubated under slow mixing overnight at 4 °C. The homogenate was subsequently incubated with magnetic beads for 1 h at RT under constant mixing, and subsequently washed according to the manufacturer’s specifications. Elution buffer was added to the tube and incubated for 10 min at RT and the supernatant-containing antigen was magnetically separated from the beads. After adding the neutralization buffer, the sample was then subjected to western blotting analysis.

### Immunoblotting analysis

Western blot was performed according to the manufacturer’s protocol. Equal amounts of protein were subjected to sodium dodecyl sulfate-polyacrylamide gel electrophoresis (SDS–PAGE) gels followed by transfer to PVDF membranes. Membranes were probed with primary antibodies overnight and then washed and incubated with horseradish peroxidase (HRP)-conjugated-secondary antibodies. Detection was performed by the enhanced chemical luminescence (ECL) method. The primary antibodies of NCOR2 (ab24551; 1:1000) was purchased from Abcam. CRBN (NBP1-91810; 1:1,000) antibody was purchased from Novus Biologicals. c-Myc (#5605; 1:1,000), IKAROS (#9034; 1:1,000), MTA1 (#5647; 1:1,000), MBD3 (#14540; 1:1,000), HDAC1 (#5356; 1:1,000), and HDAC2 (#5113; 1:1,000), antibodies were purchased from Cell Signaling Technology, Inc.

### Whole-exome sequencing

Sequencing alignment was performed using bwa (version 0.7.17) [11] with respect to human reference genome build hg19. After alignment, duplicated reads were removed with Picard (version 2.8.0, http://broadinstitute.github.io/picard/). We adopted GATK (version 4.0.0.0) [12] variant calling pipeline to call variants for each sample independently. Variants were filtered base on the recommended filtration, quality depth > 2.0, FisherStrand < 60, mapping quality > 40.0, mapping quality rank-sum test > −12.5, read position rank-sum test > −8.0. Annovar annotation tool [13] was used to perform functional annotation of filtered variants. Summaries of variants categorized according to various annotated features using in-house scripts.

### Statistical analysis

**Fig. 1:**
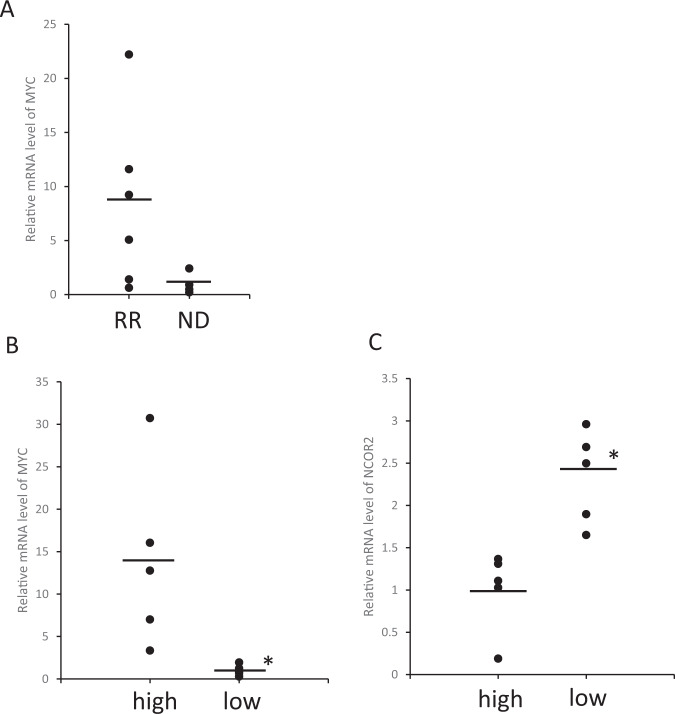
CD138-positive patient-derived multiple myeloma cells with high MYC expression correlate with low NCOR2 expression. **A** Patient-derived CD138+ cells were analyzed for transcript levels of MYC by qPCR respectively. 6 patients had relapsed (RR) MM and 4 patients were newly diagnosed (ND) MM. **B**–**C** mRNA level of MYC and NCOR2 were divided in 2 groups (high MYC and low MYC) according to MYC mRNA level. *P < 0.01.

All in vitro experiments were performed in triplicate and repeated at least 3 times and a representative experiment was selected for the figures. The statistical analysis was performed using SPSS Statistics version 25 (IBM Japan). The statistical significance of differences was determined using a two-sided paired Student *t* test with a minimal level of significance of *p* < 0.05. Error bars are representative of ±standard error of the mean (s.e.m.). The variance was similar between the groups that were statistically compared. With 5 high MYC patients and 5 low MYC patients in NCOR2 measurement group (Fig. 1C), an independent t test will have at least 80% power at the 5% significance level.

## Results

### CD138-positive patient-derived multiple myeloma cells with high MYC expression correlate with low NCOR2 expression

MYC is an oncogene that is elevated in IMiD refractory patients and associated with poor prognosis [6]. We analyzed MYC expression of patient BM-derived primary CD138-positive MM cells from four newly diagnosed (ND) and six relapsed MM patients. Gene expression analysis of MYC by RT-PCR showed that MYC was upregulated in relapsed MM patients as compared with NDMM, although not significantly so (Fig. 1A). Further, we categorized the patients based on MYC expression (Fig. 1B). We discovered that patients with high MYC expression correlated with low NCOR2 expression (Fig. 1C). NCOR2 is a nuclear receptor co-repressor (NCOR) targeting various cancer-related transcription factors. NCOR2 was previously reported to be down-regulated in MM cell lines and patient-derived myeloma cells and induces tumor cell proliferation, although the underlying mechanism is not fully understood.

### Depletion of NCOR2 confers multidrug resistance and MYC upregulation independent of CRBN

**Fig. 2:**
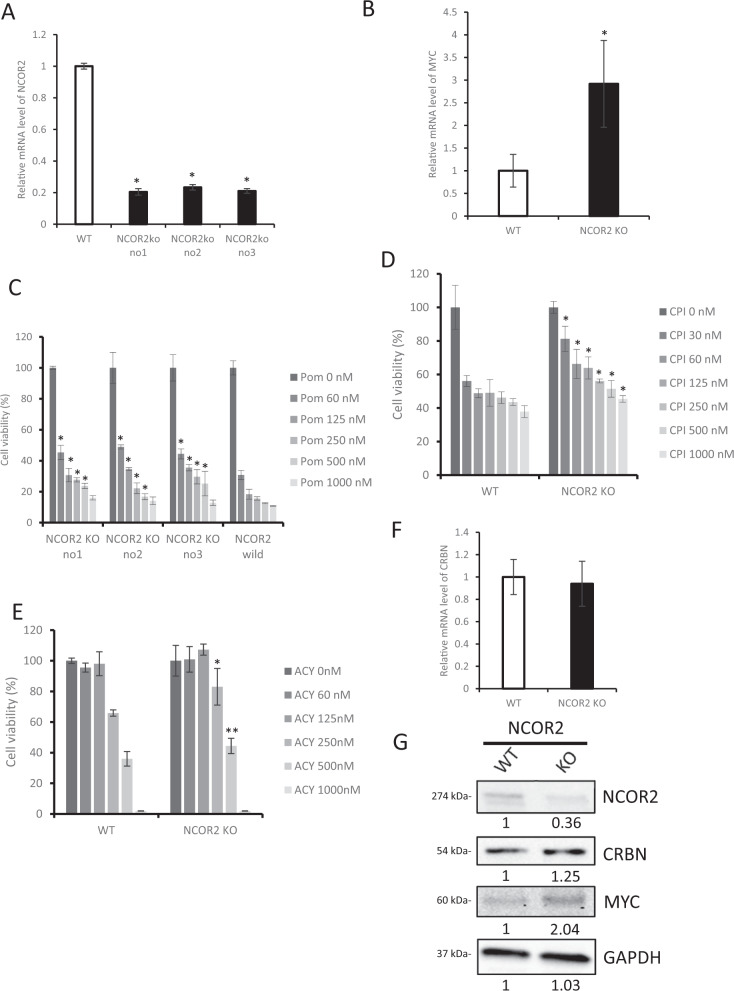
Depletion of NCOR2 confers multidrug resistance and MYC upregulation independent to CRBN. **A** Three NCOR2 knock out MM.1 s cell lines were created using CRISPR/cas9 gene modification and mRNA expression level of NCOR2 in these knock out cell lines (NCOR2 ko no1, no2, and no3) and control MM.1 s cell line (WT) were examined by qPCR. **B** mRNA expression level of MYC in NCOR2 knock out MM.1 s (KO) and control MM.1 s (WT) were examined by qPCR. **C** Three NCOR2 knock out MM.1 s (NCOR2 KO1, NCOR2 KO2, NCOR2, KO3) and control MM.1 s cell line (NCOR2 wild) were exposed to increasing concentrations of Pomalidomide for 72 h. Cell viability was assessed by CellTiter-Glo Luminescent Cell Viability Assay. **D** NCOR2 knock out MM.1 s (NCOR2 KO) and control MM.1 s cell line (NCOR2 wild) were exposed to increasing concentrations of CPI0203 for 48 h. Cell viability was assessed by CellTiter-Glo Luminescent Cell Viability Assay. **E**, NCOR2 knock out MM.1 s (NCOR2 KO) and control MM.1 s cell line (NCOR2 wild) were exposed to increasing concentrations of ACY1215 for 48 hours. Cell viability was assessed by CellTiter-Glo Luminescent Cell Viability Assay. **F**, mRNA expression level of CRBN in NCOR2 knock out MM.1 s (KO) and control MM.1 s (WT) were examined by qPCR. **G**, NCOR2 knock out MM.1 s (KO) and control MM.1 s (WT) were analyzed for protein levels of CRBN and MYC by immunoblot respectively. *P < 0.01, **P < 0.05.

To further study the underlying biological significance of NCOR2 in MM, we developed 3 NCOR2 knockout (KO) MM.1 s cell lines using CRISPR/cas9 (Fig. 2A). NCOR2 KO cell lines showed high MYC expression (Fig. 2B), similar to the results of CD138-positive MM patient cells. Based on the fact that high MYC expression leads to IMiD resistance in MM patients [6], we tested NCOR2 KO cell lines with pomalidomide by cell viability analysis. Our data demonstrated that NCOR2 KO cell lines were resistant to pomalidomide (Fig. 2C). Next, we tested the resistance of NCOR2 KO cell line with a BET inhibitor CPI0203, which has been shown to downregulate MYC [14]. Surprisingly, NCOR2 KO cell lines also demonstrated a relative resistance to CPI0203 (Fig. 2D). We report similar resistance-phenotype with NCOR2 KO cell lines when treated with HDAC inhibitor (Fig. 2E). Gene expression and protein analysis of NCOR2-KO MM.1 s confirmed the expression of CRBN in NCOR2 KO MM.1 s did not change significantly compared with control MM.1 s (Fig. 2F, G). Together, these results demonstrate that NCOR2 mediated induction of MYC is independent of CRBN, and potentially leads to multidrug resistance in MM.

### NCOR2 links to NuRD complex and downregulate MYC via CD180 inhibition

**Fig. 3:**
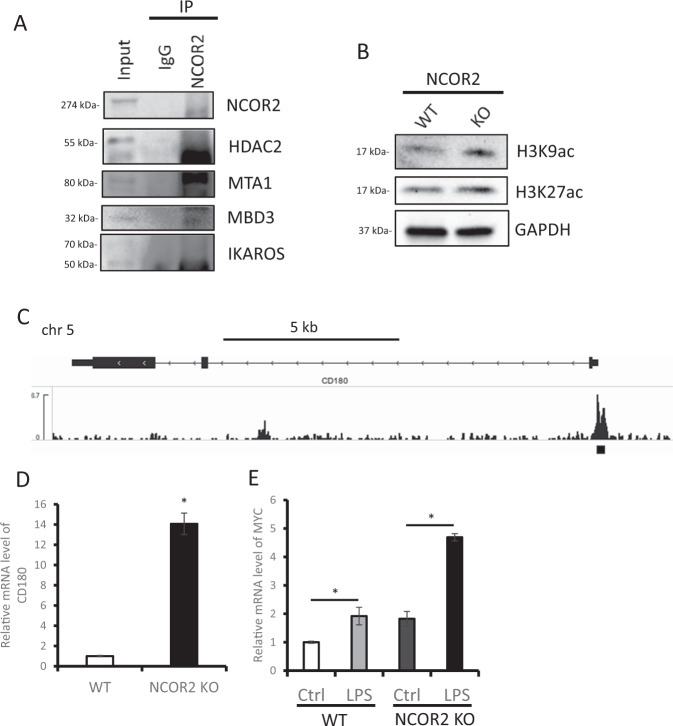
NCOR2 links to NuRD complex and downregulate MYC via CD180 inhibition. **A** Western blot analysis of co-immunoprecipitation of endogenous proteins by anti-NCOR2 in MM.1 s were analyzed. **B** Western blot analysis of H3K9ac and H3K27ac in MM.1 s and NCOR2 knock out MM.1 s were analyzed. **C** NCOR2 binding at the promoters of CD180. y-axis values represent read densities normalized to total number of reads. **D** mRNA expression level of CD180 in NCOR2 knock out cell line (KO) and control MM.1 s cell line (WT) were examined by qPCR. **E** mRNA expression level of MYC in NCOR2 knock out cell line (KO) and control MM.1 s cell line (WT) with or without LPS stimulation (10 nM, 4 h) were examined by qPCR. *P < 0.01.

NCOR2 is an established corepressor that negatively regulates gene expression coupled with the HDACs and their enhanced activity [15]. Specifically, HDACs-1,2 comprise the core components of the NuRD complex, essential for B cell development and inhibition of downstream signaling molecules along with IKAROS [16]. Direct interaction of NCOR2 with MTA1, MBD3 and HDAC2 as analyzed by co-IP established the functional interaction of the NuRD complex-IKAROS (Fig. 3A). Next, analysis of H3K9ac/H3K27ac revealed significant upregulation of acetylation in NCOR2-KO cells (Fig. 3B). Together, these data indicate that NCOR2 interacts with NuRD complex along with IKAROS and enhances the deacetylation of H3K9/H3K27, repressing downstream targets. The targets of NCOR2 were previously investigated by Hatzi et al. using ChIP-seq data for NCOR2 in human B cells [17], with no peak in the MYC-promoter, indicating indirect upregulation of MYC (data not shown). Analysis of genes that showed peak call within 2000 bases of Transcription Start Site (TSS) helped identify CD180. CD180 is a receptor of Lipopolysaccharide (LPS), directly regulated by NCOR2 (Fig. 3C), and highly upregulated in NCOR2-KO MM.1 s cells (Fig. 3D). Moreover, LPS stimulation of NCOR2-KO induced significant MYC upregulation as compared to controls (Fig. 3E). This shows that CD180 is upregulated in multidrug-resistant MM and is susceptible to LPS stimulation, and causing upregulation of MYC.

### Long term treatment with IMiD induces NCOR2 mutation and downregulation of NCOR2

**Fig. 4:**
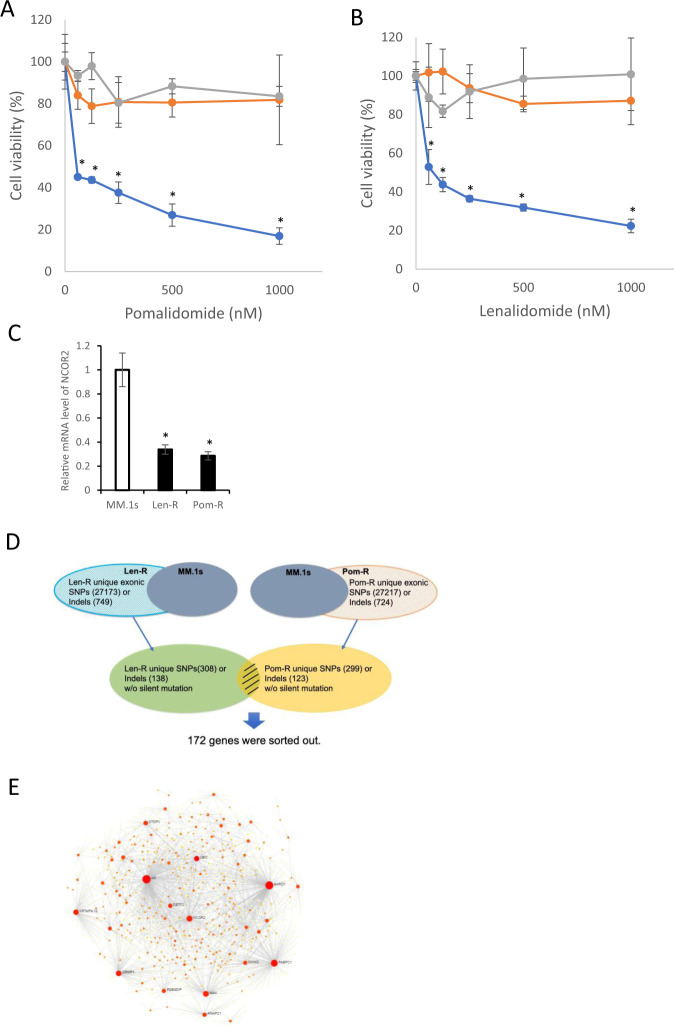
Long term treatment of IMiD induce NCOR2 mutation and downregulation of NCOR2. **A** Three myeloma cell (MM.1 s, Len-R and Pom-R) lines were exposed to increasing concentrations of Pomalidomide for 72 h. Cell viability was assessed by CellTiter-Glo Luminescent Cell Viability Assay. **B** Three myeloma cell (MM.1 s, Len-R and Pom-R) lines were exposed to increasing concentrations of Lenalidomide for 72 h. Cell viability was assessed by CellTiter-Glo Luminescent Cell Viability Assay. **C** Three myeloma cell lines were analyzed for mRNA levels of NCOR2 by qPCR respectively. **D** Whole-exome sequencing of MM.1 s and 2 resistant cell lines (Len-R and Pom-R) compared with reference sequence were performed and 172 genes with exonic mutations in both Len-R and Pom-R myeloma cells were sorted out. **E** A protein-protein interaction (PPI) network of the 172 genes demonstrated 8 genes that scored a high degree of PPI. *P < 0.01.

To examine whether NCOR2 downregulation was acquired via long term drug exposure, we established lenalidomide-resistant (Len-R) and pomalidomide-resistant (Pom-R) MM.1 s MM cell lines by exponentially growing MM.1 s in the presence of Len/Pom. Both Len-R/Pom-R showed resistance to IMiDs (Fig. 4A, B). Similar to NCOR2 KO cells, Len-R/Pom-R cell lines showed significantly decreased levels of NCOR2 (Fig. 4C). Next, we performed whole-exome sequencing (WES) of Len-R/Pom-R MM cell lines with control MM.1 s cells. Analysis of the WES data revealed more than 27,000 SNPs and 700 indel mutations in each cell line. We sorted out 172 genes harboring unique non-silent mutations in Len-R/ Pom-R MM cells (Fig. 4D, Supplemental Table S1). We then constructed a protein-protein-interaction (PPI) network by KEGG analysis (Fig. 4E), revealing 8 top genes with significantly high degree distribution. Among these 8 genes, we were able to identify NCOR2, which demonstrated the same gained exonic mutation (p.P510delinsQQP). These data suggest that long term IMiD treatment induces NCOR2 mutation and decreased level of NCOR2 expression. Additionally, we also identified CRBN, which gained two nonsense mutations (p.R418X, p.E329X) in Pom-R and 1 nonsense mutation (p.R418X) in Len-R (Supplemental Fig. S1) MM cells, validating acquired IMiD resistance in Len-R and Pom-R by the gain of point mutations in CRBN.

### IMiD resistant cell lines show multidrug resistance via MYC elevation

**Fig. 5:**
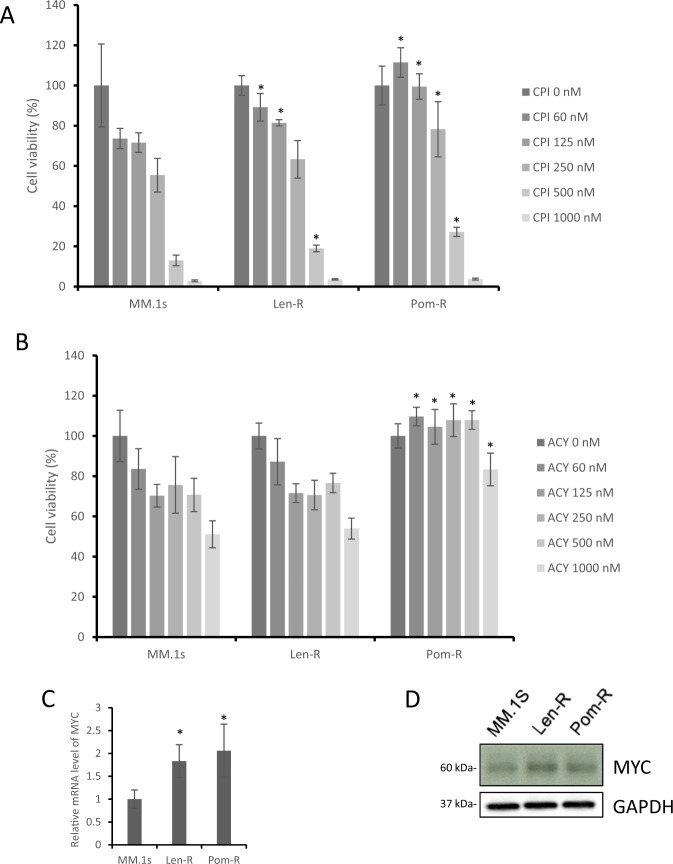
IMiD resistant cell lines show multidrug resistance via MYC elevation. **A** Three myeloma cell (MM.1 s, Len-R and Pom-R) lines were exposed to increasing concentrations of CPI0203 for 48 h. Cell viability was assessed by CellTiter-Glo Luminescent Cell Viability Assay. **B** Three myeloma cell (MM.1 s, Len-R and Pom-R) lines were exposed to increasing concentrations of ACY1215 for 48 h. Cell viability was assessed by CellTiter-Glo Luminescent Cell Viability Assay. **C** Three myeloma cell lines were analyzed for mRNA levels of MYC by qPCR respectively. **D** Three myeloma cell lines were analyzed for protein levels of MYC by immunoblot respectively. *P < 0.01.

To further examine the IMiD resistant cell lines harboring NCOR2 mutation, we tested Len-R/Pom-R cell lines with CPI0203/ACY1215 as compared to control cells. Pom-R MM cells showed significant resistance to both agents similar to that seen in the NCOR2 KO cell line (Fig. 5A, B), while Len-R MM cells showed significant resistance to CPI0203. Both Len-R/Pom-R cell lines showed significantly increased levels of MYC expression at the mRNA and protein levels, compared to the parental cell line (Fig. 5C, D) suggesting that MYC upregulation contributes to multidrug resistance.

### High MYC expression is induced by NCOR2-CD180 pathway, independent of CRBN in IMiD resistant human cells

**Fig. 6:**
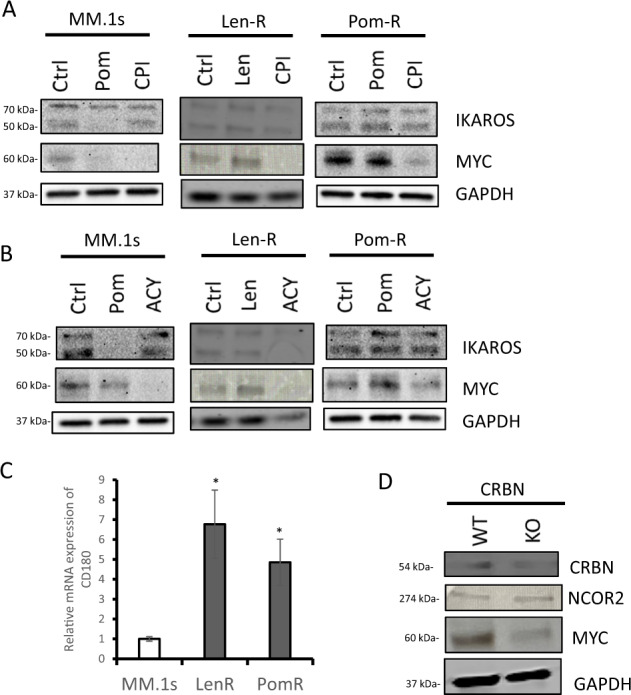
High MYC expression is induced by NCOR2-CD180 pathway, independent of CRBN in IMiD resistant human cells. **A** IKAROS and MYC protein levels were analyzed by Western blot in MM.1 s, Len-R and Pom-R after a 24-h treatment with 1000 nM of Pomalidomide (Pom), 1000 nM of CPI-0203 (CPI) versus control (Ctrl). **B** IKAROS and MYC protein levels were analyzed by Western blot in MM.1 s and Pom-R after a 24-h treatment with 1000 nM of Pomalidomide (Pom), 1000 nM of ACY1215 (ACY) versus control (Ctrl). **C** mRNA expression level of CD180 in MM.1 s and IMiD resistant MM.1 s cell lines (Len-R and Pom-R) were examined by qPCR. **D**, CRBN, NCOR2 and MYC protein levels were analyzed by Western blot in CRBN ko 293 T cell line and control 293 T cell line. *P < 0.01.

As mentioned above, Len-R/Pom-R cell lines acquired the same nonsense mutation of CRBN and gained IMiD resistance after long exposure of IMiD. Western blot analysis of CRBN in Len-R/Pom-R cell lines revealed reduced protein level of CRBN compared with parental MM.1 s cells (Supplemental Fig. S2). Furthermore, a similar nonsense mutation is known to cause a structural change in CRBN and lead to resistance of IMiDs [18]. These data demonstrate that the acquired CRBN mutation in these resistant cell lines can cause IMiD resistance. However, CRBN mutation alone cannot explain the multidrug resistance via MYC upregulation in these cells. To clarify whether the high MYC expression in IMiD resistant cell lines is regulated by CRBN malfunction, we examined the expression of IKAROS and MYC, both downstream targets of CRBN, following exposure to the BET inhibitor or HDAC inhibitor. Both the BET inhibitor (CPI0203) and the HDAC inhibitor (ACY1215) completely inhibited MYC, analyzed by western blot analysis in MM.1 s when directly compared with Pom-R cell line, where it was partially inhibited (Fig. 6A, B). We did not see any changes in the IKAROS levels, which is the direct target of CRBN mediated-degradation by IMiDs when treated with CPI0203/ACY1215 (Fig. 6A, B). These results indicate upregulation of MYC in IMiD resistant cell lines are blocked by BET inhibitor or HDAC inhibitor via CRBN-IKAROS independent pathway. The results of NCOR2-KO cell lines suggest that NCOR2-NuRD complex can directly regulate CD180, resulting in MYC upregulation. Similar to NCOR2-KO cell lines, CD180 was highly upregulated in Len-R/Pom-R (Fig. 6C). Furthermore, we demonstrated that knockdown of CRBN did not alter the expression of NCOR2 and even blocked the expression of MYC (Fig. 6D). These data again indicate that high MYC expression in Len-R/Pom-R is induced by NCOR2-CD180 pathway independent of CRBN.

## Discussion

In this study, we investigated the underlying mechanism for IMiD resistance independent of CRBN in MM patients. We identified NCOR2 as a gene that gains loss-of-function mutation by continuous exposure to IMiDs. Surprisingly, downregulation of NCOR2 caused multidrug resistance including against IMiDs by enhancing the expression of CD180, the direct target of NCOR2, with consequent MYC upregulation. Although NCOR2 mutations have not been described as common events in newly diagnosed patients [19], acquired mutations of NCOR2 after IMiD treatment have not been fully investigated in the past underscoring the need for further studies. Reduced level of NCOR2 in MM patients has been reported to promote the proliferation of MM cells via upregulation of JAG2, a NOTCH ligand overexpressed in MM [20]. We could not prove the direct binding of NCOR2 to JAG2 promotor by ChIP-seq data (data not shown), but this report coincides with our data showing the relation between low NCOR2 level and the progression of MM.

Although our study is limited due to the small patient sample cohort, we further investigated the significance of NCOR2 in MM and demonstrated that low NCOR2 expression correlated with high MYC expression. High MYC expression in tumor cells of MM patients has been shown to corelate with poor prognosis [21], suggesting the correlation of low NCOR2 and poor prognosis. Furthermore, these data show that low NCOR2 has a potential to be harnessed as a biomarker of drug resistance and may enable early decision making for change of treatment from IMiDs.

CD180 is a LPS receptor similar to Toll-like receptor 4 (TLR4) and is reported to promote oncogenesis and tumor growth. We showed that CD180 could be a direct target of NCOR2, altering the expression of MYC in MM. Kikuchi et al. [22] reported the underlying CD180 mediated drug-resistance pathways in MM, regulated by IKAROS. Additionally, they report stimulation of CD180 by LPS secreted from the tumor microenvironment leads to MM growth. This study aligns with our findings of CD180 regulation by NCOR2 along with IKAROS. In order to elucidate the precise molecular mechanism of NCOR2-CD180-MYC pathway, experiments of overexpressing NCOR2 in IMiD resistant cell lines is ongoing.

Overcoming drug resistance caused by NCOR2 silencing is therapeutically challenging in the clinic. However, direct inhibition of MYC using protein targeting chimeric molecules (PROTAC) may be a strategy that requires exploration. Hatakeyama et al. reported a PROTAC with MAX, which forms a heterodimer with MYC, and E3 ligase recognition site [23]. This PROTAC physically interacts with MYC and promotes the proteasomal degradation of MYC. Additionally, several novel drugs have been invented to inhibit MYC [5], including a dual acting inhibitor, CUDC-907, blocking-HDAC and PI3K simultaneously inhibiting MYC [24]. These may be candidates to overcome drug resistance in future studies.

In conclusion, we report NCOR2 mediated drug resistance in MM occurs by MYC upregulation in MM, and is independent of CRBN. Low NCOR2 expression presents a potential new biomarker for IMiD refractory patients.

## Supplementary information

Supplemental Figure legends

Supplemental figure1 and figure2

Supplemental table1
