# Supplementary material for: Low NCOR2 levels in multiple myeloma patients drive multidrug resistance via MYC upregulation
Source: Blood Cancer J. 2021 Dec 4;11(12):194. doi: 10.1038/s41408-021-00589-y (PMC8643354; doi:10.1038/s41408-021-00589-y)
Supplement: Supplementary file 1 — Supplemental Figure legends [file 41408_2021_589_MOESM1_ESM.docx]

**Supplemental Information**

**Supplemental Figures**

**Supplemental Figure S1**: **Len-R and Pom-R gained nonsense mutations of CRBN.**

**Supplemental Figure S2**: **Protein level of CRBN in Len-R and Pom-R MM.1s cells is decreased compared with parental MM.1s.**

CRBN protein levels were analyzed by Western blot in MM.1s, Len-R and Pom-R.

**Supplemental Tables**

**Supplemental Table S1: Len-R and Pom-R acquired 172 genes harboring unique non-silent mutations.**
