# Supplementary material for: Low NCOR2 levels in multiple myeloma patients drive multidrug resistance via MYC upregulation
Source: Blood Cancer J. 2021 Dec 4;11(12):194. doi: 10.1038/s41408-021-00589-y (PMC8643354; doi:10.1038/s41408-021-00589-y)
Supplement: Supplementary file 2 — Supplemental figure1 and figure2 [file 41408_2021_589_MOESM2_ESM.pptx]

## Slide 1
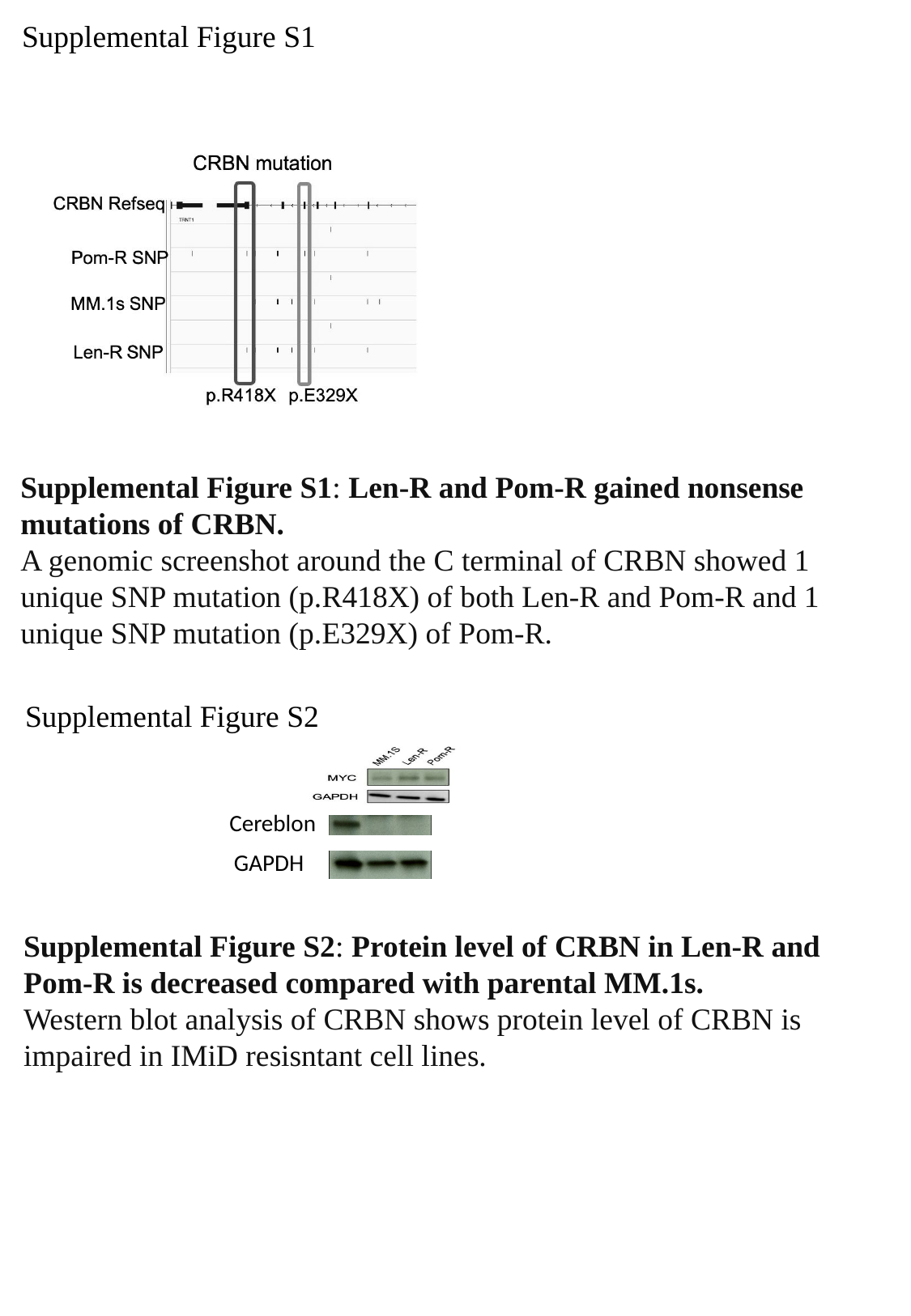

Supplemental Figure S1
Supplemental Figure S1: Len-R and Pom-R gained nonsense mutations of CRBN.
A genomic screenshot around the C terminal of CRBN showed 1 unique SNP mutation (p.R418X) of both Len-R and Pom-R and 1 unique SNP mutation (p.E329X) of Pom-R.
Supplemental Figure S2
Cereblon
GAPDH
Supplemental Figure S2: Protein level of CRBN in Len-R and Pom-R is decreased compared with parental MM.1s.
Western blot analysis of CRBN shows protein level of CRBN is impaired in IMiD resisntant cell lines.
