# Supplementary material for: Low NCOR2 levels in multiple myeloma patients drive multidrug resistance via MYC upregulation
Source: Blood Cancer J. 2021 Dec 4;11(12):194. doi: 10.1038/s41408-021-00589-y (PMC8643354; doi:10.1038/s41408-021-00589-y)
Supplement: Supplementary file 3 — Supplemental table1 [file 41408_2021_589_MOESM3_ESM.pptx]

## Slide 1
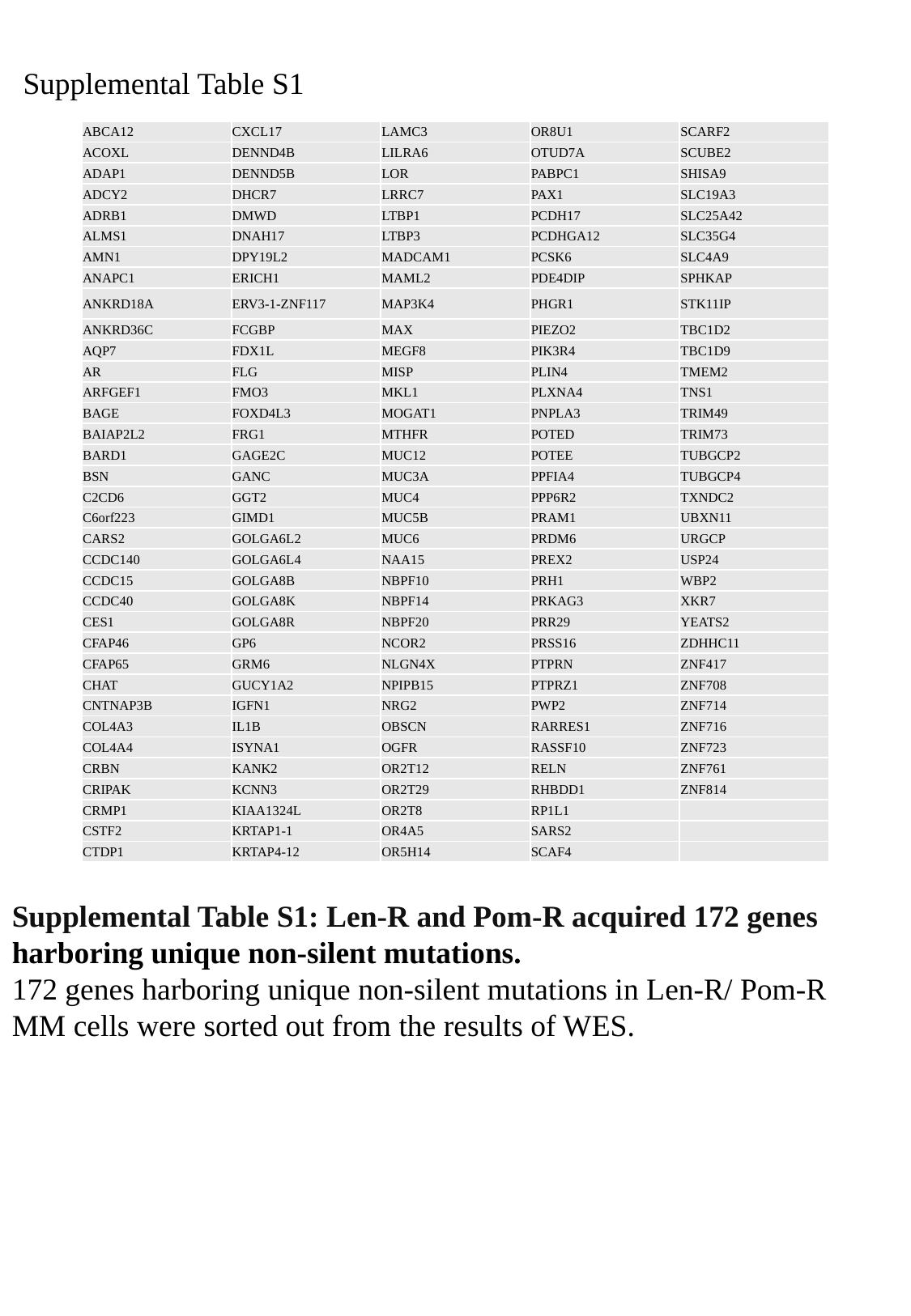

Supplemental Table S1
| ABCA12 | CXCL17 | LAMC3 | OR8U1 | SCARF2 |
| --- | --- | --- | --- | --- |
| ACOXL | DENND4B | LILRA6 | OTUD7A | SCUBE2 |
| ADAP1 | DENND5B | LOR | PABPC1 | SHISA9 |
| ADCY2 | DHCR7 | LRRC7 | PAX1 | SLC19A3 |
| ADRB1 | DMWD | LTBP1 | PCDH17 | SLC25A42 |
| ALMS1 | DNAH17 | LTBP3 | PCDHGA12 | SLC35G4 |
| AMN1 | DPY19L2 | MADCAM1 | PCSK6 | SLC4A9 |
| ANAPC1 | ERICH1 | MAML2 | PDE4DIP | SPHKAP |
| ANKRD18A | ERV3-1-ZNF117 | MAP3K4 | PHGR1 | STK11IP |
| ANKRD36C | FCGBP | MAX | PIEZO2 | TBC1D2 |
| AQP7 | FDX1L | MEGF8 | PIK3R4 | TBC1D9 |
| AR | FLG | MISP | PLIN4 | TMEM2 |
| ARFGEF1 | FMO3 | MKL1 | PLXNA4 | TNS1 |
| BAGE | FOXD4L3 | MOGAT1 | PNPLA3 | TRIM49 |
| BAIAP2L2 | FRG1 | MTHFR | POTED | TRIM73 |
| BARD1 | GAGE2C | MUC12 | POTEE | TUBGCP2 |
| BSN | GANC | MUC3A | PPFIA4 | TUBGCP4 |
| C2CD6 | GGT2 | MUC4 | PPP6R2 | TXNDC2 |
| C6orf223 | GIMD1 | MUC5B | PRAM1 | UBXN11 |
| CARS2 | GOLGA6L2 | MUC6 | PRDM6 | URGCP |
| CCDC140 | GOLGA6L4 | NAA15 | PREX2 | USP24 |
| CCDC15 | GOLGA8B | NBPF10 | PRH1 | WBP2 |
| CCDC40 | GOLGA8K | NBPF14 | PRKAG3 | XKR7 |
| CES1 | GOLGA8R | NBPF20 | PRR29 | YEATS2 |
| CFAP46 | GP6 | NCOR2 | PRSS16 | ZDHHC11 |
| CFAP65 | GRM6 | NLGN4X | PTPRN | ZNF417 |
| CHAT | GUCY1A2 | NPIPB15 | PTPRZ1 | ZNF708 |
| CNTNAP3B | IGFN1 | NRG2 | PWP2 | ZNF714 |
| COL4A3 | IL1B | OBSCN | RARRES1 | ZNF716 |
| COL4A4 | ISYNA1 | OGFR | RASSF10 | ZNF723 |
| CRBN | KANK2 | OR2T12 | RELN | ZNF761 |
| CRIPAK | KCNN3 | OR2T29 | RHBDD1 | ZNF814 |
| CRMP1 | KIAA1324L | OR2T8 | RP1L1 | |
| CSTF2 | KRTAP1-1 | OR4A5 | SARS2 | |
| CTDP1 | KRTAP4-12 | OR5H14 | SCAF4 | |
Supplemental Table S1: Len-R and Pom-R acquired 172 genes harboring unique non-silent mutations.
172 genes harboring unique non-silent mutations in Len-R/ Pom-R MM cells were sorted out from the results of WES.
